# Lessons Learned From a Program to Reduce Diabetes Risk Among Low-Income Hispanic Women in a Community Health Clinic

**DOI:** 10.3389/fendo.2020.489882

**Published:** 2021-01-08

**Authors:** Nangel M. Lindberg, Sonia Vega-López, Erin S. LeBlanc, Michael C. Leo, Victor J. Stevens, Sara Gille, Mayra Arias-Gastelum, Richard Meenan

**Affiliations:** ^1^ Kaiser Permanente Center for Health Research, Portland, OR, United States; ^2^ College of Health Solutions, Arizona State University, Phoenix, AZ, United States; ^3^ Faculty of Nutrition Sciences, University of Sinaloa, Culiacán, Mexico

**Keywords:** women—diseases, intervention—behavioral, disparities (health), diabetes mellitus type 2, Hispanic (demographic)

## Abstract

**Background:**

The Diabetes Prevention Program (DPP) and Look AHEAD studies demonstrated that modest weight loss and increased physical activity can significantly reduce the incidence of diabetes among overweight individuals with prediabetes. However, these studies involved costly interventions, all of which are beyond the reach of most real-world settings serving high-risk, low-income populations. Our project, De Por Vida, implemented a diabetes risk-reduction intervention for Hispanic women in a Federally Qualified Health Center and assessed the program’s efficacy. This report describes the methodology used to develop and implement De Por Vida, the cultural adaptations made, the community–academic partnership formed to carry out this program, and the barriers and challenges encountered through the implementation process.

**Methods:**

Our goal was to translate the DPP and Look AHEAD programs into an intervention to prevent diabetes and reduce diabetes complications among high-risk Hispanic women at a federally qualified health center in Hillsboro, Oregon, where more than half of clinic patients are Spanish-speaking, and nearly all live in poverty. This randomized clinical trial targeted overweight Spanish-speaking women at risk for, or diagnosed with, type 2 diabetes. We developed a 12-month behavioral diabetes risk-reduction intervention that was responsive to the cultural practices of the Hispanic population and that could be implemented in low-income clinical settings. Study planning and implementation involved close collaboration among the clinic leadership, a research team from the Kaiser Permanente Center for Health Research, and Arizona State University.

**Discussion:**

Creating a fully informed partnership between research and clinical institutions is the first step in successful cooperative research projects. The adoption of a bidirectional, rather than a top-down, approach to communication between researchers and health-care providers, and between clinic management and the clinic frontline staff, gave the research study team crucial information about barriers, constraints, and needs that clinic staff experienced in implementing the program. This allowed clinic management and front-line clinic staff to play an active role in study implementation, identifying problem areas, and collaborating in finding practical solutions.

**Clinical Trial Registration:**

www.clinicaltrials.gov, NCT03113916.

## Introduction

Hispanics, comprising more than 56 million people in the United States, are the largest minority group in the country (1). This demographic group is among the most vulnerable to obesity-related disease and disability (2, 3). Obesity prevalence is increasing at particularly alarming rates among Hispanic women (4); this, along with the risk that Hispanic ethnicity poses for the development of type 2 diabetes (T2D), places Hispanic women at high risk for developing the disease (5, 6). As shown in the Hispanic Community Health Study, by the time they reach age 70, nearly half of Hispanic women will have diabetes, with higher prevalence rates for those with lower levels of education or income (6).

Our project, De Por Vida, was a randomized clinical trial assessing the efficacy of a culturally tailored diabetes risk-reduction intervention for Hispanic women, implemented in a Federally Qualified Health Center (FQHC). A detailed description of the study protocol has been published elsewhere (7). This report describes the culturally-coherent intervention that was used, the community-academic partnership we formed with the clinic where the study was implemented, the barriers and challenges we encountered during implementation, and lessons learned over the course of the study (7–9).

Weight loss, along with diet and physical activity (PA), is the first-line treatment to prevent and control diabetes (10–12). For individuals diagnosed with T2D, weight loss is particularly important because many diabetes medications result in weight gain (11, 13). Lifestyle interventions resulting in modest weight loss can prevent or delay diabetes onset by 33 to 68% among individuals with pre-diabetes (14, 15) and can result in clinically meaningful reductions in cardiovascular disease (CVD) risk factors and greater reduction in medication use among diabetes patients (16–19).

The Diabetes Prevention Program (DPP) and the Look AHEAD Study were both multi-site randomized clinical trials that successfully demonstrated that modest weight loss and increased physical activity can significantly reduce the incidence of diabetes among overweight individuals with prediabetes (20) and improved biomarkers of glucose and lipid control, and improved quality of life in overweight individuals with type 2 diabetes (21). However, these studies involved costly interventions, including reliance on individual sessions, meal replacements, or the use of registered dietitians, behavioral psychologists, and exercise specialists, all of which are beyond the reach of most real-world clinics serving high-risk, low-income populations (20, 21).

Like the DPP and Look AHEAD interventions, De Por Vida targeted weight loss through dietary change and increased physical activity with the goal of reducing diabetes risk and complications. We specifically targeted overweight Spanish-speaking women at risk for or diagnosed with T2D. Our goal was to develop a diabetes risk-reduction intervention that was responsive to the cultural practices of the Hispanic population (22), and that could be implemented in clinical settings serving this population.

There were critical differences between the De Por Vida program and the DPP; whereas the DPP was not designed as an “intervention that could be translated for use in community settings” (23), our intervention was specifically designed to be used in a community health center. Unlike the DPP, our intervention did not involve individual sessions, but was group-based, and the interventionists were not master’s-level dietitians, behavioral interventionists, or exercise physiologists, but medical assistants.

The purpose of this paper is not to provide a detailed protocol, nor to present study results, but rather to offer lessons learned in the implementation of a clinical trial that tested an intervention in a real-life community clinic. Because few studies have reported on the challenges of doing this kind of work, we believe this information will prove useful for future implementation studies.

## Methods and Design

### Setting

The project was carried out in Hillsboro, Oregon, a diverse community that is more than 30% Hispanic. We implemented the program at the Virginia Garcia Memorial Health Center (VGMHC), a FQHC serving 45,000 patients, 56% of whom are Spanish-speaking individuals of Mexican origin. Nearly 98% of patients live in poverty (earning less than 200% of the federal poverty level). Approximately 71% of patients are covered by Medicaid, Medicare, Oregon Health Plan, or private insurance. Services are provided on a sliding-scale basis and no one is turned away for inability to pay.

### Study Design

We conducted a randomized controlled clinical trial (RCT) to test a lifestyle intervention for obese and overweight Hispanic women with or at risk for prediabetes or T2D. The aims of the intervention were to reduce body weight and waist circumference. Secondary aims were to improve markers of glycemic control (fasting blood glucose and HbA1c) and cardiovascular risk (serum lipid profile), diet, and physical activity. Data from the previous De Por Vida pilot study (24) were used to estimate the sample size. The number of subjects needed to detect a significant in change in weight between the arms at 12 months was 52, with a power of.80 and alpha of .05. Assuming a 66% retention rate, as observed in the De Por Vida pilot study, we recruited 100 women per arm. Using A1c data from Hispanic participants in the DPP studies, we calculated that 60 subjects per arm would allow us to detect a difference in % HbA1c change over time of 0.26 in HbA1c with statistical power of 0.26 and an alpha level of .05.

**Figure 1:**
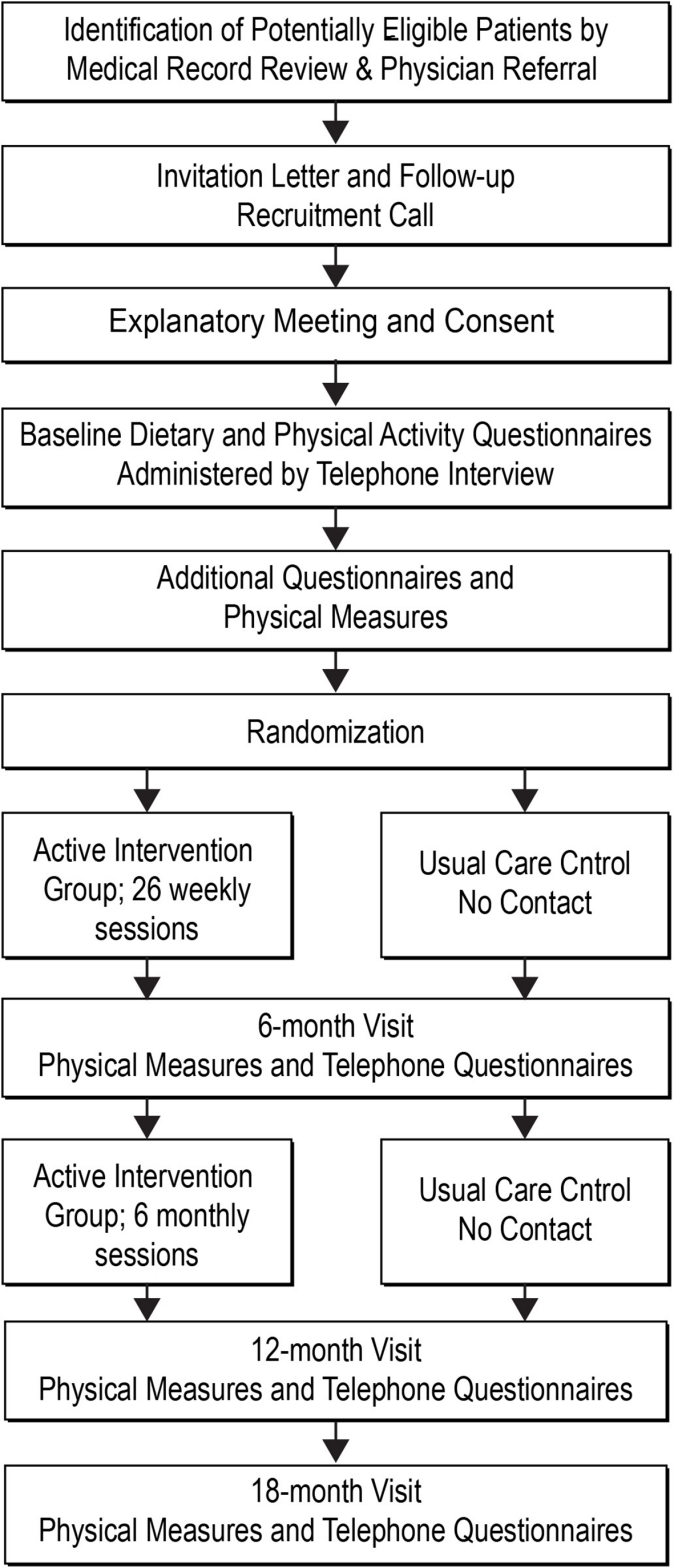
Project design.

Trial participants were randomly assigned to one of two conditions, with 100 participants per condition: (1) Usual care control, or (2) a culturally tailored behavioral intervention. Randomization was conducted using a computerized block randomization scheme and was stratified by baseline BMI (30–34, 35–39, 40 or more) and age (18–45, 46 and above). Outcome assessments with participants from both conditions were conducted at baseline, 6, 12, and 18 months (see **Figure 1**). Recruitment was conducted in seven cohorts of approximately 30 randomized participants each. A usual care control condition was chosen to ensure participants who did not receive the intervention continued receiving standard medical care at the clinic.

### Study Implementation and Management

Study planning and implementation involved a collaboration among the clinic leadership, a research team from the Kaiser Permanente Center for Health Research (KPCHR) and an academic institution, Arizona State University (ASU). The Institutional Review Boards of all three organizations approved the study.

The Principal Investigator (PI) (Lindberg) was a Mexican clinical psychologist and KPCHR investigator with an extensive background in behavioral interventions, obesity research, and cultural adaptation of interventions. The PI had primary responsibility for the day-to-day operation of the study; thus, she was in close contact with clinic staff and administrators at all stages of the project. The lead nutritionist (Vega-López), a Co-Investigator (Co-I) on the study team, was a Mexican-born Associate Professor of Nutrition at ASU with extensive expertise in the dietary habits of Hispanic individuals and in developing community-based interventions to prevent and control chronic disease. The nutritionist Co-I collaborated in the design of intervention materials, outcome measures, and data collection procedures. The clinic medical director (Turner), also a Co-I, oversaw overall study operations within the clinic, and collaborated in the development and planning of recruitment, randomization, intervention, and data-collection phases.

The three partners in this collaboration communicated formally through weekly, and later monthly, in-person or telephone meetings at the clinic, and also informally through regular telephone calls and emails. Daily management of study activities and data collection was facilitated by a web-based, password-protected tracking system kept on a server at KPCHR. The tracking system could only be accessed by authorized study personnel at the clinic, KPCHR, and ASU.

Throughout the trial’s planning phase, recruitment and randomization, the project employed a top-down communications approach, with weekly meetings attended by the study PI, study project manager, the VGMHC medical director, and other clinic managers. Additionally, the clinic program managers held weekly meetings with the five medical assistants they supervised. These medical assistants were critical to the success of our study, because they functioned as recruiters and interventionists.

### Startup: Adapting to Challenges

Before recruitment started, the study PI and the clinic medical director hosted a one-hour kick-off meeting to which every clinic staff member was invited. During the kick-off meeting, which was conducted during a time the clinic was closed to patients to maximize staff attendance, the PI and medical director introduced the study staff and explained the purpose of the study, study duration, the recruitment and enrollment process, the intervention, and follow-up assessments. Staff were encouraged to ask questions and were given the PI’s contact information.

Soon after recruitment began, it became apparent that the project was interfering with clinic routine. For example, because some exam rooms were used for research project data collection, there was a shortage of exam rooms for patient care. In addition, some clinic staff not directly involved in the study became overburdened; for example, clinic managers in charge of coverage schedules for medical assistants found they had added coverage needs and insufficient personnel, and clinic receptionists and offsite clinic telephone operators had to field questions about a study they knew little or nothing about.

Our study needed, but did not yet have, full buy-in from the clinic’s front-line staff—telephone operators, receptionists, and particularly medical assistants, who would serve as study interventionists and would thus be critical to our study’s success. These staff members were tasked with additional jobs related to our study, but they were not given extra time to do these extra tasks. Offsite call-center operators had not been included in the kick-off meeting and lacked the basic information they needed to answer questions about the study.

We decided to conduct an informational meeting with the offsite call center personnel to explain the study’s purpose and describe the process of recruitment, enrollment, and follow-up assessment. Because there was rapid turnover of call center staff, we repeated the informational meeting every 6 months. Realizing that there was a communications gap between the front-line staff, the research team, and upper clinic management, we decided to adopt a bidirectional approach—both between the clinic and the research team, and between the upper clinic management and the administrative and front-line staff—for all subsequent stages of the project. This included asking medical assistants, as well as representatives of other clinic departments involved in and impacted by the study (medical providers, front desk, call center, and clinic workflow staff) to participate in weekly study meetings to help identify and solve existing or emerging problems.

Frequent points of contact between all levels of the research team and clinic staff resulted in improved understanding by both the clinic and the research teams of each other’s objectives, tasks, needs, and constraints, and this in turn resulted in refinement of the research procedures to better address both parties’ needs, including needs for funding. For example, the study team allocated funds for the clinic to hire additional personnel to fully cover the clinical responsibilities of those medical assistants who were study interventionists. We also provided them with a study-dedicated cell phone to reduce the burden on clinic telephone operators and streamline communication between study participants and study personnel.

In spite of improved communication, some clinic-specific issues created barriers for smooth implementation. Specifically, the turnover of clinic staff, including mid-level administrative staff (clinic supervisors, managers, administrators), and entry-level employees (call center operators, medical assistants, receptionists), led to a lack of continuity in administrative tasks. This problem was often compounded by the clinic not informing the research team of upcoming clinic staffing changes impacting the research project. For example, over a period of 3 years, the research team worked with 4 different clinic managers, 5 different nursing/MA staff supervisors, and over 5 different individuals in charge of submitting clinic invoices to KPCHR (the awardee). In some cases, neither the study PI nor the study project manager were informed that clinic staff of strategic importance for project implementation (e.g., the Clinic Program Director) were no longer at the clinic.

Our study was also impacted by important differences in priorities, function, and culture between research-oriented and clinic-based team members. Researchers were focused on following protocols and were used to a culture of open communication and transparency regarding needed resources. By contrast, clinic staff were focused primarily on immediate patient management and providing care, were accustomed to making do with limited resources, and did not always communicate about issues not directly related to patient care.

### Recruitment

Although recruitment methods included direct referrals by clinic physicians, materials posted in exam rooms, and patient word of mouth, our primary recruitment method began with the clinic’s electronic medical record (EMR), which generated a mailing list of potentially eligible patients approximately every 2 months. We queried the EMR using study inclusion criteria: (1) Having been seen at the clinic within the last 18 months, (2) Spanish-speaking, (3) age 18 and older, (4) female, (5) body mass index (BMI) ≥27 kg/m^2^, and (6) presence of either diabetes or prediabetes in the patient problem list, or presence of A1c ≥5.7% or fasting blood glucose (FBG) ≥100 mg/dL, or history of hypertension, hyperlipidemia, gestational diabetes, or family history of diabetes. Due to different pregnancy and lactation dietary requirements and patient safety, the query excluded patients identified as pregnant or within 12 months post-partum. Based on preliminary numbers gleaned from initial data pulls, and out of concern that we may not have sufficient numbers for recruitment, we decided to lower our BMI eligibility criterion from our initially planned 30 to 27 kg/m^2^.

Because of the central role that “confianza”, a mixture of familiarity and trust (25), plays in interpersonal relationships among Hispanic people, we decided that, in addition to the PI, study interventionists would recruit participants. The two interventionists were medical assistants, both native Mexican Spanish-speakers, who had each worked at the clinic for more than 15 years and were familiar to patients and their families.

Patients who were flagged in the EMR as potentially eligible were mailed a recruitment letter written in Spanish and signed by the study’s lead physician. The letter briefly described the study, explained why the patient had been identified as potentially being able to benefit, and invited interested patients to call for additional information. The two interventionists/recruiters returned these calls during business hours as well as on evenings and weekends, as per patient preference. Patients who did not respond to the recruitment letter also received telephone calls. Because most patients were familiar with the medical assistants, the calls usually started with a brief informal conversation about the patient’s and her family’s health, family life events, and recent events in the local community or in their communities of origin before moving on to providing information about the study. Patients expressing interest in the study were briefly screened over the phone to confirm potential eligibility; once confirmed, our recruiters invited these women to an in-person information session at the clinic.

### Information Sessions and Consent

At the group information sessions, one recruiter/interventionist explained the study, including what random assignment involved and why it was necessary, what the two study arms involved, that participation was voluntary, potential risks and benefits of the study, and answered questions. Patients who chose to continue with enrollment received two copies of the study consent form. Recruiters read and explained the form, described next steps, and answered questions. Patients who signed the informed consent (taking one copy home) were instructed to wait for a telephone call from a study interviewer to proceed with the baseline data collection and randomization process.

### Baseline Data Collection

Master’s-level trained bilingual interviewers called consented participants to schedule a 45-min telephone interview. During the interview, participants completed the interviewer-administered Southwestern Food Frequency Questionnaire (SWFFQ) (26–28) and the General Practice Physical Activity Questionnaire (GPPAQ) (29). At the end of the telephone interview, participants were scheduled for a data collection visit at the clinic. At that visit, trained medical assistants measured weight, waist circumference, and height. A fasting finger stick test was used to assess lipid profile, blood glucose, and HbA1c.

While waiting for lab results, participants completed a paper-and-pencil survey which consisted of: Short Form-12 quality of life questionnaire (30, 31), Barriers to Healthy Eating Questionnaire (BHEQ) (32), questions to assess health literacy and (33) numeracy skills (34), and the language-literacy based Brief Acculturation Scale for Hispanics (35). If participants needed help completing the questionnaires, the medical assistant administered them as an interview. After completion, the PI told the patients their weight and the results of their blood test and explained that their medical provider would also receive the lab results. Following confirmation of their intention to participate, patients were randomized and informed of their group assignment. Patients without a diabetes diagnosis but with HbA1c ≥6.5% and/or FBG ≥126 mg/dL results were scheduled for a follow-up appointment with their provider.

### Study Conditions

Immediately after baseline data collection but before randomization, all participants were given a digital bathroom scale, a set of measuring cups and spoons, a pedometer, and two Spanish-language booklets on nutrition and weight loss (36). Individuals were then randomized to either enhanced usual care (EUC) or to the De Por Vida culturally tailored lifestyle intervention condition.

### Lifestyle Intervention

We developed De Por Vida as a group-based intervention (26 weekly sessions followed by 6 monthly sessions), incorporating content from the Diabetes Prevention Program (DPP) (37), Look AHEAD (38), and PREMIER (39) trials. All nutrition recommendations were congruent with current American Diabetes Association (ADA) guidelines (40) and included increasing consumption of non-starchy vegetables and whole grains, and reducing intake of sugar, refined carbohydrates, starches, and saturated fats. Participants were encouraged to consume several small meals during the day, rather than one or two large meals, avoid skipping meals, and increase satiety by consuming more vegetables, dietary fiber, and water. Based on current guidelines (41), we recommended that participants gradually increase their physical activity to at least 150 min per week or 10,000 steps per day.

#### Cultural Adaptations

The cultural adaptations implemented in the program have been described in detail elsewhere (24) and were developed in a manner consistent with the growing consensus regarding cultural adaptation of lifestyle intervention programs (42, 43). We adapted intervention materials for language, literacy and numeracy skills, product preference, food choices, meal schedules, and holidays and special events relevant to Hispanic populations. Intervention content incorporated topics central to the immigrant experience, including financial difficulties, discrimination, acculturative stress, pressures to adopt “American-style” diet habits and values, interfamily conflict, and disruption of family and social networks.

The intervention also addressed traditional beliefs regarding health and food, such as a hot-cold concept of humoral medicine (44), the use of nopales (cactus pads) or chia seeds for glycemic control (45), and widespread beliefs in Mexican culture that adding lime juice to foods “burns the fat”, or that soft tortillas are “more fattening” than toasted ones (46).

#### Intervention Content

The protocol included basic nutrition information, hands-on opportunities to learn and practice food measurement for portion control, and instruction and practice in goal setting and self-monitoring to help participants reach their goals in weight loss, physical activity, and dietary change. Intervention materials were tailored to the predominantly Mexican background and low-literacy needs of the study participants; we focused on providing a practical understanding of diabetes and fostering attitudes and behaviors consistent with diabetes prevention and glycemic control (e.g., establishing short-, medium-, and long-term lifestyle change goals, identifying barriers, and coping with challenges for behavior change). Materials included a booklet that classified foods frequently consumed by Hispanic individuals into three major groups (proteins, carbohydrates, and fats), and included calorie content for each food. Foods were color-coded with a traffic light (green, yellow, and red) according to their saturated fat content and glycemic index (e.g., butter is coded red because it is high in saturated fat).

Consistent with most weight loss interventions (47), we encouraged participants to keep food diaries to monitor and control their food intake. In addition to standard food journaling—i.e., writing down the specific food item, amount consumed, and its estimated caloric content—participants were taught an alternative method of journaling in which they tallied their servings of fruits, vegetables, protein, carbohydrates, and water each day. Participants turned in their weekly food journals at every session, and interventionists reviewed the journals and returned them at the following session along with feedback.

Per participants’ requests, twice per month we conducted food preparation demonstrations to teach participants how to modify traditional dishes to be in line with ADA dietary recommendations (e.g., using whole wheat tortillas instead of flour tortillas). Our intervention encouraged participants to engage in physical activity; however, beyond a session explaining the benefits of physical activity, exercise was not a central focus of the intervention sessions. Participants expressed an interest in group exercise; in response to this, one of the interventionists led a weekly drop-in 15-min, low-impact exercise segment to salsa music that took place before the scheduled session. To maintain rapport and engagement, we called participants weekly to remind them of each upcoming session.

#### Group Structure and Format

Each 90-min session included these components: (1) brief exercise segment; (2) weigh-in; (3) group sharing and problem solving regarding goals and action plans from the previous week; (4) discussion of a weight-management information topic related to behavior change—such as self-monitoring, relapse prevention, stress management, nutrition, and/or physical activity; (5) food demonstration or practice activities—such as food measuring or role-playing; (6) goal setting and action plans for the following week (shared with the group). Attendance at each session varied, with an average of five participants per session. There were no penalties for missed sessions.

#### Interventionist Training

We developed an intervention manual to guide delivery of the sessions. The protocol was implemented and delivered by the PI and the two Mexican-born Spanish-speaking female MAs who were well known to most patients. The interventionists were trained on motivational counseling principles, and extensively trained on delivering the intervention protocol, including principles of behavior change, basic nutrition and exercise, group management skills, and strategies to facilitate behavior change and manage resistance. The PI trained the interventionists.

### Follow-Up Assessments

At 6-, 12-, and 18-month post-randomization, study staff masked to group assignment called participants to schedule a follow-up telephone interview during which participants completed interviewer-administered surveys. A complete list of measures is presented in **Table 1**. In addition to the specified surveys, during each follow-up interview we asked open-ended questions regarding possible intervention-related adverse events (e.g., hospitalizations, visits to urgent care, or emergency rooms) in the prior 6 months.

**Table 1:** List of Survey Instruments and Their Frequency**.

Instrument	Baseline	6-month	12-month	18-month
Southwest Food Frequency Questionnaire (26–28)	195	152	139	141
Barriers for Healthy Eating (32)	195	152	139	141
General Practice Physical Activity Questionnaire (29)	195	152	139	141
Client Satisfaction Questionnaire (48)	n/a	152	139	141
Short Form-12 Quality of Life (30, 31, 49)	195	152	139	141
Literacy and Numeracy questions (34)	195	n/a	n/a	n/a
Language Based Brief Acculturation Scale for Hispanics (35)	195	n/a	n/a	n/a

**Assessments completed by participants in both arms of the study.

After each telephone interview, a clinic visit was scheduled for the following week to measure weight, waist circumference, fasting lipid profile, HbA1c, and FBG. At the completion of each of the 6-, 12-, and 18-month visits, participants were given a $25, $30, and $35 gift card to a local supermarket, respectively. The incentives were identical for individuals in both arms.

### Final Enrollment and Baseline Characteristics of Participants

**Figure 2:**
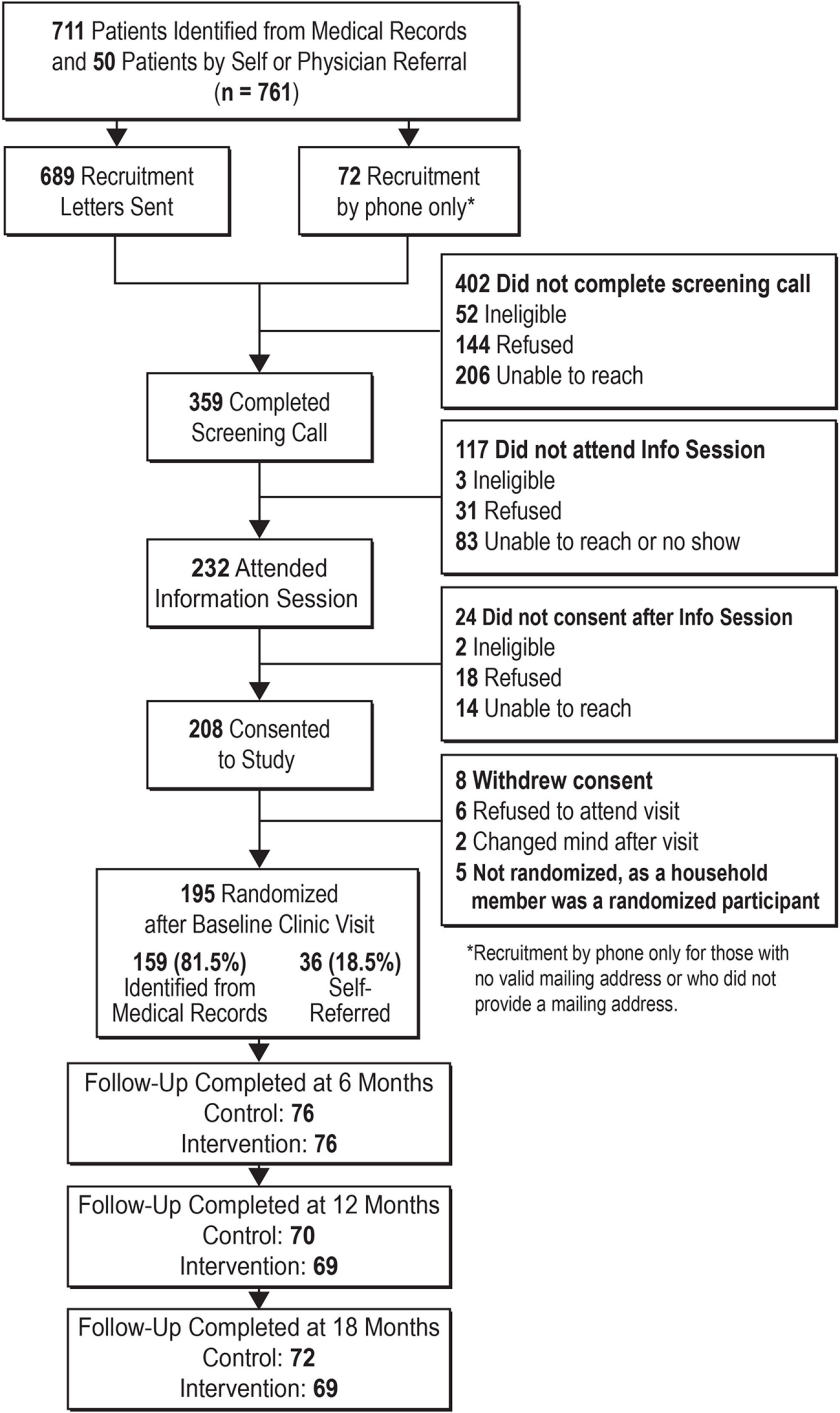
Consort diagram.

As shown in the study consort diagram (**Figure 2**), of the 761 participants that were referred to the study or identified *via* medical records, 359 completed the screening call, 232 attended the information session, and 208 consented to the study. Eight declined participation. Five women had a member of their household previously randomized; to prevent violation of the assumption of independence, instead of being randomized, these women were assigned to the same arm as their household member and their data was not collected for analysis. In total, 195 were randomized to the study. The majority (96%) of participants were Mexican-born with the remaining being immigrants from Guatemala (n = 6) and El Salvador (n = 2). Participants’ baseline characteristics are presented in **Table 2**. The study population was female, with a mean age ± SD of 44 ± 9.9 years, with ages ranging from 18 to 73 years. Mean body weight was 86.7 ± 16.6 kg, BMI was 36.5 ± 6.5 kg/m^2^, waist circumference was 115.4 ± 13.5 cm. Complete laboratory data were available for 192 participants [HbA1c = 6.5 ± 1.5% (47.7 ± 15.9 mmol/mol) FBG = 134.4 ± 44.0 mg/dL (7.5 ± 2.9 mmol/L)]. Data from the EMR showed that 68 (35%) had a diagnosis of type 2 diabetes, 40 (21%) had a diagnosis of prediabetes, and 87 participants (45%) were at risk for diabetes but had no diagnoses of either type 2 diabetes or prediabetes.

**Table 2:** Participant Baseline Characteristics Mean (SD).

Characteristics	Total	Control	Intervention
Age	44.0 years (9.9)	43.4 years (9.5)	44.5 years (10.3)
Body weight (n = 195)	86.7 kg (16.6)	86.3 kg (16.4)	87.2 kg (16.8)
BMI (n = 195)	36.5 kg/m^2^ (6.5)	36.5 kg/m^2^ (6.6)	36.6 kg/m^2^ (6.5)
Waist circumference (n = 194)	115.4 cm (13.5)	115.4 cm (13.6)	115.4 cm (13.5)
HbA1c (n = 192)	6.5% (1.5%)	6.5% (1.5%)	6.5% (1.5%)
FBG (n = 192)	134.4 mg/dL (44.0)	135.8 mg/dL (46.7)	133.1 mg/dL (41.6)

## Discussion

Research conducted within clinical settings offers the possibility of measuring or accessing relevant data, examining and addressing patient concerns, informing the development of accessible and pragmatic interventions, and improving patient care, especially for patients who are frequently hard to reach and underrepresented in research. Yet it can also present unique and difficult challenges, including discrepant goals in research vs. clinical practice, the need for additional staff or space to conduct clinical research tasks, difficulties integrating research practices into clinic routines, clinical staff’s lack of experience with field research requirements, and the clash of different professional cultures (50). These challenges are even greater in clinical settings with limited financial resources, which may experience frequent changes in clinic staff positions and responsibilities.

Several limitations of our study must be noted, including the narrow range of demographic characteristics of the study participants—female, mostly Mexican immigrants with low income and limited literacy. Similarly, our experience may not generalize to non-FQHC clinical settings that may operate under different leadership and funding mechanisms. Additionally, with the exception of biologic and anthropometric data, all the data derived from this study relied on participants’ self-report. Lastly, when considering implementing this type of intervention program in community-based institutions providing services in underserved areas, the cost of adding such a program would be a serious financial challenge.

This study also has important strengths. First, the De Por Vida program was tested using the gold standard randomized controlled trial design. Second, the study was implemented in a real-life setting and involved an underrepresented population at high risk for diabetes. Third, because we had a small number of interventionists with close oversight from the PI, the intervention was delivered with high fidelity and consistency. Finally, we utilized deep cultural adaptation strategies to make De Por Vida better suited for its target population.

The De Por Vida study established a community-academic partnership to carry out a diabetes risk reduction project, translating a research project originally tested in a research setting into a FQHC clinical setting. Whereas De Por Vida incorporated some elements from the DPP and the Look AHEAD programs, there are critical differences between those research programs and our clinic-based intervention. Both DPP and Look AHEAD were large-scale efficacy studies conducted in highly structured controlled research environments, which sought to establish a link between weight loss and diabetes risk reduction. By contrast, De Por Vida tested the efficacy of a culturally tailored intervention while navigating the hurdles of implementing this intervention in a clinical setting that serves a vulnerable low-income immigrant population.

Despite the fact that Hispanics are less likely to participate in research compared to other racial/ethnic groups (51), and that recruitment of this population remains challenging (52), De Por Vida achieved its target recruitment goal. We believe our success was due to key factors: First, recruiters were clinic staff who were known to and highly trusted by clinic patients. Additionally, while our recruitment contact was not formally designed as culturally tailored or individual-centered, the fact that the contact was loosely scripted, and took place between people who shared the same cultural background, and who often had a long-standing relationship of trust, made this communication both culturally coherent and highly effective. The active involvement of front-line clinic staff in all stages of the project was also a critical element.

Creating a fully informed partnership between research and clinical institutions is the first step in successful cooperative research projects. The adoption of a bidirectional, rather than a top-down, approach to communication between researchers and health-care providers, and between clinic management and the clinic frontline staff, gave the research study team crucial information about barriers, constraints, and needs that clinic staff experienced in implementing the program. This shift allowed clinic management and front-line clinic staff to play an active role in study implementation, identifying problem areas and collaborating in finding practical solutions.

De Por Vida demonstrated that a diabetes-risk reduction intervention can be successfully implemented in a community clinic serving a low-income Hispanic population. De Por Vida recruited patients diagnosed with, or at-risk for diabetes, and carried out the intervention and study assessments completely in Spanish, demonstrating that the process of translating research into clinical settings serving vulnerable populations is possible—although not without numerous challenges. Our experience—the challenges we encountered, and the bidirectional approach we adopted to learn about problems and overcome them—can serve other research teams wishing to implement studies in real-world clinical settings.

## Data Availability Statement

The original contributions presented in the study are included in the article. Further inquiries can be directed to the corresponding author.

## Ethics Statement

The studies involving human participants were reviewed and approved by Kaiser Permanente Northwest Institutional Review Board and the Arizona State University Institutional Review Board. The patients/participants provided their written informed consent to participate in this study.

## Author Contributions

NL, SV-L, EL, VS, ML, and RM participated in conceptualization and design of the study. NL, MA-G, EL, and SG participated in conducting the study. NL, SV-L, EL, and VS drafted or critically revised the initial drafts of the manuscript. All authors contributed to the article and approved the submitted version.

## Funding

This work was supported exclusively by the National Institutes of Health/National Institute of Diabetes and Digestive and Kidney Diseases 1R01DK099277 (Lindberg, PI). Beyond providing funding for this project, the study sponsor played no role in, or authority over, the design, development, or implementation of this project, the data collection or analyses, or the development or submission of any reports emanating from this study, including the present manuscript.

## Conflict of Interest

The authors declare that the research was conducted in the absence of any commercial or financial relationships that could be construed as a potential conflict of interest.
